# Methodological quality assessment of genetic studies on brain arteriovenous malformation related hemorrhage: A cross-sectional study

**DOI:** 10.3389/fgene.2023.1123898

**Published:** 2023-03-23

**Authors:** Junhao Jiang, Zhuo Qin, Junxia Yan, Junyu Liu

**Affiliations:** ^1^ Hunan Normal University School of Medicine, Changsha, China; ^2^ Department of Epidemiology and Health Statistics, XiangYa School of Public Health, Central South University, Changsha, China; ^3^ Hunan Provincial Key Laboratory of Clinical Epidemiology, XiangYa School of Public Health, Central South University, Changsha, China; ^4^ Interventional Medical Center, Hunan Province People’s Hospital (The First Affiliated Hospital of Hunan Normal University), Changsha, China; ^5^ Department of Pharmacology, Kyoto University Graduate School of Medicine, Kyoto, Japan

**Keywords:** brain arteriovenous malformation, intracranial hemorrhage, genetics, methodological quality, rupture

## Abstract

**Objectives:** Rupture of a brain arteriovenous malformation (bAVM) can cause intracranial hemorrhage and severe clinical outcomes. At present, the mechanisms of bAVM-related hemorrhage are poorly understood. This study aimed to summarize the potential genetic risk factors for bAVM-related hemorrhage and appraise the methodological quality of existing genetic studies on bAVM-related hemorrhage using a cross-sectional design.

**Methods:** A systematic literature search was conducted on genetic studies associated with bAVM-related hemorrhage published in PubMed, Embase, Web of Science, China National Knowledge Internet, and Wangfang databases, up to November 2022. Subsequently, a cross-sectional study was performed to describe the potential candidate genetic variants of bAVM associated with risk of hemorrhage and to evaluate the methodological quality of the identified studies using the Newcastle–Ottawa quality assessment scale and Q-genie tool.

**Results:** Of the 1811 records identified in the initial search, nine studies met the filtering criteria and were included. Twelve single nucleotide polymorphisms (SNPs), including *IL6* rs1800795, *IL17A* rs2275913, *MMP9* rs9509, *VEGFA* rs1547651, and *EPHB4* rs314353*,* rs314308, and rs314313, were associated with bAVM-related hemorrhage. However, only 12.5% of the evaluated SNPs showed statistical power> 0.80 (*α* = 0.05). Methodological quality assessment revealed significant flaws in the designs of the included studies, such as less reliable representativeness of recruited individuals, short follow-up periods in cohort studies, and less comparability between groups of hemorrhagic and non-hemorrhagic patients.

**Conclusion:** *IL1B*, *IL6*, *IL17A*, *APOE*, *MMP9*, *VEGFA* and *EPHB4* were potentially associated with bAVM-related hemorrhage. The methodological designs of the analyzed studies required improvement in order to obtain more reliable results. Regional alliances and rare disease banks need to be established to recruit large numbers of bAVM patients (especially familial and extreme-trait cases) in a multicenter, prospective cohort study with an adequate follow-up period. Furthermore, it is important to use advanced sequencing techniques and efficient measures to filter candidate genetic variants.

## 1 Introduction

The most common and severe manifestation of a brain arteriovenous malformation (bAVM) is its rupture, which is also the leading cause of intracranial hemorrhage in children and young adults. High pressure blood flow from the feeding arteries of the bAVM floods directly to the draining veins through the malformed nidus, causing the development of abnormal shear stress due to lack of capillary structure within the anomalous nidus, ultimately resulting in its rupture (Rutledge et al., 2014). Past observational studies have reported a 1%–3% annual incidence of bAVM-related hemorrhage in unruptured and untreated patients, whereas the reported risk was much higher in individuals with ruptured bAVM(2). Current treatments, including microsurgery, endovascular embolization, and stereotactic radiosurgery aim to reduce the risk of hemorrhage and eradicate existing lesions. Although microsurgery offers the advantage of a higher rate of complete obliteration and elimination of bAVM-related hemorrhage compared to the other treatments, craniotomy is a highly traumatic procedure resulting in a longer hospitalization as well as substantial morbidity and mortality during the perioperative period (van Beijnum et al., 2011; Derdeyn et al., 2017). Thus, it is imperative to identify risk factors for bAVM rupture as early as possible.

Prior hemorrhage has been reported to be associated with a higher rate of subsequent hemorrhage as a strongly predictive factor (Chen et al., 2020). Existing evidence suggests that angioanatomic features of bAVM, including large size, deep venous drainage, few draining veins, and coexisting arterial aneurysm, contribute to its rupture (Krithika and Sumi, 2021). Several studies have investigated and discovered genetic variants of inflammation- or angiogenesis-related genes that could potentially influence bAVM rupture by accelerating growth and modifying lesion behavior to promote disease pathogenesis (Pawlikowska et al., 2004; Achrol et al., 2006; Pawlikowska et al., 2006; Kim et al., 2009; Weinsheimer et al., 2009; Gong et al., 2011; Li et al., 2012; Sun et al., 2012; Delev et al., 2017). However, due to the low prevalence and incidence of bAVM, most genetic studies on bAVM recruited small samples of patients and were prone to selection bias, resulting in inconsistent results. In addition, different research designs may yield conflicting results and may have varying methodological quality. Therefore, existing genetic studies on bAVM cannot always be considered as a reliable source of evidence. A well-performed research that provides reliable and high-quality information can help medical practitioners in improving their understanding of the nature of a particular disease as well as assist them in making appropriate treatment decisions. Before accepting and using any scientific evidence, healthcare professionals, medical managers, health policymakers, and even patients should evaluate the methodological quality of the referred studies.

The present study performed a cross-sectional survey to summarize the genetic factors influencing the risk of hemorrhage associated with bAVM and evaluate the methodological rigor of the included studies using the Newcastle–Ottawa quality assessment scale (NOS) and Q-genie tool. Our aim was to summarize the current information regarding genetic risk of bAVM-related hemorrhage, discuss potential research directions, and provide insights into how to improve the methodological quality in future studies investigating the risk of bAVM-related hemorrhage or hemorrhagic stroke caused by other diseases.

## 2 Materials and methods

### 2.1 Eligibility criteria

This study was registered with PROSPERO (CRD42021258353). All included articles were case-control or cohort studies recruiting individuals of any ethnic group and focused on the genetic risk factors associated with bAVM-related hemorrhage, using the methods of candidate gene association studies (CGAS), genome-wide association studies (GWAS) or whole exome sequencing (WES). Individuals diagnosed with bAVM based on a recognized criteria were recruited and divided into ruptured and unruptured groups (Atkinson et al., 2001). Only original papers with accurate and sufficient genotyping data that could allow the calculation of odds ratios (ORs) and 95% confidence intervals (95% CIs) were included in the analysis. Conference abstracts, reviews, meta-analyses, protocols, case reports, and animal studies were excluded. Whenever there were duplicate or overlapping papers published by the same researcher(s), the most up-to-date version was included for evaluation.

### 2.2 Literature search strategy

We identified potential eligible studies using the search terms (“Cerebral AVM” or “cerebral arteriovenous malformation” or “brain AVM” OR “brain arteriovenous malformation”) and (“gene” or “Variation” or “polymorphisms” or “SNPs”) across five electronic databases: PubMed, Embase, Web of Science, China National Knowledge Internet (CNKI) and Wanfang Data. The search was limited to published citations in English or Chinese. Thereafter, potentially relevant studies that were not obtained in the initial searches were manually retrieved from the references of the candidate papers.

### 2.3 Data extraction

The required information (e.g., author, publication year, study design, studied genes, and SNPs) was extracted from the selected studies. When genotype frequencies of variants could not be obtained from the published papers, the risk allele frequencies of SNPs were utilized to estimate the number of cases per genotype category and calculate the OR (95% CI) using STATA 14.0 (Stata Corporation, College Station, TX, USA).

### 2.4 Methodological quality assessment

Statistical power (1-β) was calculated according to the genetic models used in the original studies *via* a two-sided Z test, using an online software (http://powerandsamplesize.com/) to evaluate the quality of studies, with the type I error rate (*α*) set at 5%. The methodological quality assessment of all included studies was mainly based on the NOS (Stang, 2010) and Q-genie tool (version 1.1) (Sohani et al., 2015). NOS is a validated appraisal tool for non-randomized studies, with eight items categorized into three dimensions: selection, comparability, and outcome (in cohort studies) or exposure (in case-control studies). A score ≥6 is regarded as high quality. The Q-genie tool (version 1.1) was used to assess the quality of the genetic association studies. It is comprised of 11 items scored from 0–7, and a total score ≤35 indicates poor quality.

Study screening, data extraction, and methodological quality assessment were independently completed by two authors, and if there was a disagreement during the process, the third senior investigator resolved the issue through re-evaluation and discussion.

## 3 Results

### 3.1 Literature selection and characteristics of included studies

**FIGURE 1:**
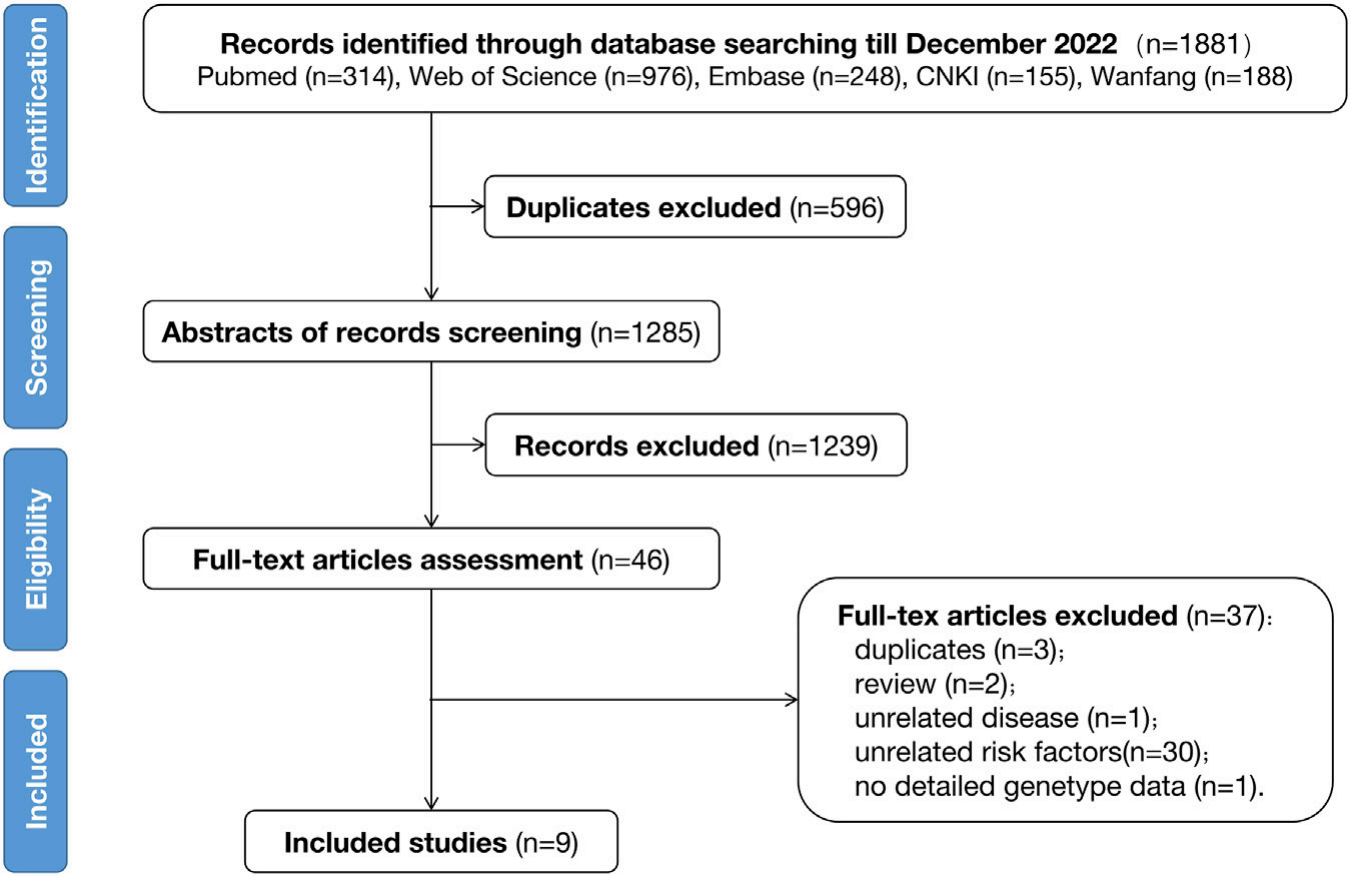
Flow diagram of the study selection process.

The initial search indicated 1881 records through a single database check until November 2022. Among these, nine studies met the eligibility criteria and were included in the final analysis. This was followed by a manual reference-list screening; however, no additional studies were found to satisfy the filtering criteria. The detailed procedure of literature selection followed in this study is displayed in Figure 1.

Six case-control studies and three cohort studies were identified, incorporating 1214 bAVM individuals from North America, Europe, and China (Table 1). The case-control studies divided patients into two groups based on the presence or absence of hemorrhage, and genotyping was performed to test the association between SNPs and bAVM rupture. In these studies (Pawlikowska et al., 2004; Weinsheimer et al., 2009; Gong et al., 2011; Li et al., 2012; Sun et al., 2012; Delev et al., 2017), a total of 984 cases had been recruited, including 529 with hemorrhage and 455 without hemorrhage. In contrast, the cohort studies relied on a prospective follow-up of the included bAVM patients until a new intracranial hemorrhage event occurred. These studies attempted to identify the association between genetic variants and the risk of new rupture during the natural process of bAVM. Furthermore, all three cohort studies (Achrol et al., 2006; Pawlikowska et al., 2006; Kim et al., 2009) belonged to the same research team, which initially recruited 237 non-hemorrhagic and 173 hemorrhagic cases. Less than 25% of the patients were followed-up for over 2 years, at the end of which twenty-seven patients had experienced bAVM rupture and new intracranial hemorrhage events. All included studies were CGAS, including four single-center studies and four multi-center studies. The last study did not mention the source of patient recruitment.

**TABLE 1:** Summary of the included genetic studies on brain arteriovenous malformation related hemorrhage.

**Gene**	**Study (Year)**	**Country**	**Language**	**Involved medical center**	**Design**	**Follow-up** **(year)**	**SNP Selection**	**Sample size**		**SNPs**	**Chromosome position, alleles, amino acid change**	**Gene function**
								**ICH**	**non-ICH**			
**Genes involving in inflammatory pathway**												
*IL6*	Pawlikowska et al. (2004)	USA	English	Single	Case-control	-	Literature review, Unigene and dbSNP database searches	73	107	rs1800795	chr7:22727026, C>G, -	Coding a cytokine functioning in inflammation and maturation of B cells.
	Achrol et al. (2006)	USA	English	Two	Cohort	0.3	NA	18	262			
	Pawlikowska et al. (2004)	USA	English	Single	Case-control	-	Literature review, Unigene and dbSNP database searches	73	107	rs1800796	chr7:22726627, G>C, -	
	Achrol et al. (2006)	USA	English	Two	Cohort	0.3	NA	18	262			
*IL10*	Pawlikowska et al. (2004)	USA	English	Single	Case-control	-	Literature review, Unigene and dbSNP database searches	73	107	rs1800896	chr1:206773552, T>C, -	Coding a cytokine mainly produced by monocytes, taking effects in immunoregulation and inflammation.
*TNF*	Pawlikowska et al. (2004)	USA	English	Single	Case-control	-	Literature review, Unigene and dbSNP database searches	73	107	rs361525	chr6:31575324, G>A, -	Coding a multifunctional proinflammatory cytokine, involved in cell proliferation, differentiation, apoptosis, lipid metabolism, and coagulation.
	Achrol et al. (2006)	USA	English	Two	Cohort	0.3	NA	18	262			
	Pawlikowska et al. (2004)	USA	English	Single	Case-control	-	Literature review, Unigene and dbSNP database searches	73	107	rs1800629	chr6:31575254, G>A, -	
	Achrol et al. (2006)	USA	English	Two	Cohort	0.3	NA	18	262			
*APOE*	Pawlikowska et al. (2006)	USA	English	Two	Cohort	0.3	NA	18	266	rs429358	chr19:45411941, T>C, p.C130R	Coding a major apoprotein of the chylomicron essential for the catabolism of triglyceride-rich lipoprotein.
										rs11542041	chr19:45411947, C>T, p.R158C	
*IL1B*	Kim et al. (2009)	USA	English	Two	Cohort	3.1	Putative functional effect and previous associated phenotypes	27	383	rs1143627	chr2:112836810, C>T, -	Coding a mediated cytokine produced by macrophages, involved in cell proliferation, differentiation, and apoptosis.
										rs16944	chr2:112837290, C>T, -	
										rs1143634	chr2:112832813, T>C, -	
*IL17A*	Li et al. (2012)	China	Chinese	NA	Case-control	-	NA	30	23	rs2275913	chr6:52186235, G>A, -	Coding a cytokine produced by activated T cells, eliciting crucial impacts on innated immune defenses.
*MMP9*	Sun et al. (2012)	China	English	Two	Case-control	-	HapMap	181	130	rs3918241	chr20:46007096, T>A, -	Coding a protein in matrix metalloproteinase family, playing roles in the breakdown of extracellular matrix in physilogical and also disease processes.
										rs1805088	chr20:46008985, C>T, p.A20V	
										rs17576	chr20:46011586, A>G, p.Q279R	
										rs3918254	chr20:46011752, C>T, -	
										rs3787268	chr20:46013092, G>A, -	
										rs17577	chr20:46014472, G>A, p.R668Q	
										rs13925	chr20:46016326, G>A, -	
										rs9509	chr20:46016514, T>C, -	
										rs17035945	chr3:12153128, C>T, -	
										rs3755724	chr3:12159406, C>T, -	
	Sun et al. (2012)	China	English	Single	Case-control	-	HapMap	181	130	rs11923383	chr3:12162637, A>G, -	
**Genes involving in angiogenesis pathway**												
*ANGPT2*	Pawlikowska et al. (2004)	USA	English	Single	Case-control	-	Literature review, Unigene and dbSNP database searches	73	107	rs3020221	chr8:6521242, C>T, p.245=	Coding an antagonist of angiopoietin 1, implicated in the inflammation- and angiogenesis- related signaling pathways.
*FLT4*	Pawlikowska et al. (2004)	USA	English	Single	Case-control	-	Literature review, Unigene and dbSNP database searches	73	107	rs448012	chr5:180619344, G>C, p.H890Q	Coding a tyrosine kinase receptor for vascular endothelial growth factors, involved in lymphangiogenesis.
										rs1130379	chr5:180612606, C>T, p.R1146H	
*KDR*	Pawlikowska et al. (2004)	USA	English	Single	Case-control	-	Literature review, Unigene and dbSNP database searches	73	107	rs1870377	chr4:55106807, T>A, p.Q472H	Also called VEGFR, coding a tyrosine kinase receptor and the main mediator of endothelial proliferation, migration and other biological function induced by VEGF.
										rs2034964	chr4:55110484, C>T, p.D392N	
*TIE2*	Pawlikowska et al. (2004)	USA	English	Single	Case-control	-	Literature review, Unigene and dbSNP database searches	73	107	rs682632	chr9:27183465, A>C, p.Q346P	Coding a tyrosine kinase receptor, binding its ligand angiopoietin-1 to mediate the signaling in embryonic vascular development.
										rs3837240	chr9:27109316, dupG, -	
										rs10967719	chr9:27108812, G>T, -	
*EPHB4*	Weinsheimer et al. (2009)	USA	English	Two	Case-control	-	HapMap	56	90	rs314346	chr7:100800895, C>T, -	Coding a Ephrin receptor, binding its ligand Ephrins to mediate numerous developmental process including nervous system and angiogenesis.
										rs314353	chr7:100808522, A>G, -	
										rs2230585	chr7:100812975, G>A, -	
										rs144173	chr7:100818628, A>G, -	
										rs314308	chr7:100823256, C>T, -	
										rs2250818	chr7:100824534, G>A, -	
										rs314313	chr7:100825743, T>C, -	
										rs2247445	chr7:100829641, G>A, -	
*VEGFA*	Pawlikowska et al. (2004)	USA	English	Single	Case-control	-	Literature review, Unigene and dbSNP database searches	73	107	rs699947	chr6:43768652, A>C, -	Coding the vascular endothelial growth factor A, a heparin-binding protein, inducing endothelial proliferation and migration in vessels in both both physiological and pathological angiogenesis.
	Gong et al. (2011)	China	English	Single	Case-control	-	HapMap	181	130	rs1547651	chr6:43762907, A>T, -	
										rs2010963	chr6:43770613, C>G, -	
										rs1413711	chr6:43772941, T>C, -	
										rs833069	chr6:43774842, T>C, -	
										rs3024994	chr6:43775770, C>T, -	
										rs3025010	chr6:43779840, T>C, -	
										rs3025030	chr6:43782850, G>C, -	
										rs3025035	chr6:43783622, C>T, -	
										rs3025039	chr6:43784799, C>T, -	
*TGFβ1*	Li et al. (2012)	China	Chinese	NA	Case-control	-	NA	30	23	rs1800469	chr19:41354391, A>G, -	Coding a ligand transforming growth factor-β, regulating cell proliferation, differentiation and growth, including vascular endothelial cells.
*TGFβR2*	Li et al. (2012)	China	Chinese	NA	Case-control	-	NA	30	23	rs3087465	chr3:30605668, A>G, -	Coding a transmembrane protein forming a complex with TGF-βR1 and then binding its ligand TGF-β to regulate gene transcription related to cell proliferation, differentiation and other biological function.
*NOTCH4*	Delev et al. (2017)	German	English	Single	Case-control	-	HapMap	64	65	rs443198	chr6:32222629, A>G, -	Coding a transmembrane protein and a menber of NOTCH family, playing a role in vascular, renal and hepatic development.
										rs915895	chr6:32222440, T>C, -	

SNP, single nucleotide polymorphisms; OR, odd ratio; 95% CI,95%confidence interval; NA, not available.

### 3.2 Genetic characteristics

**FIGURE 2:**
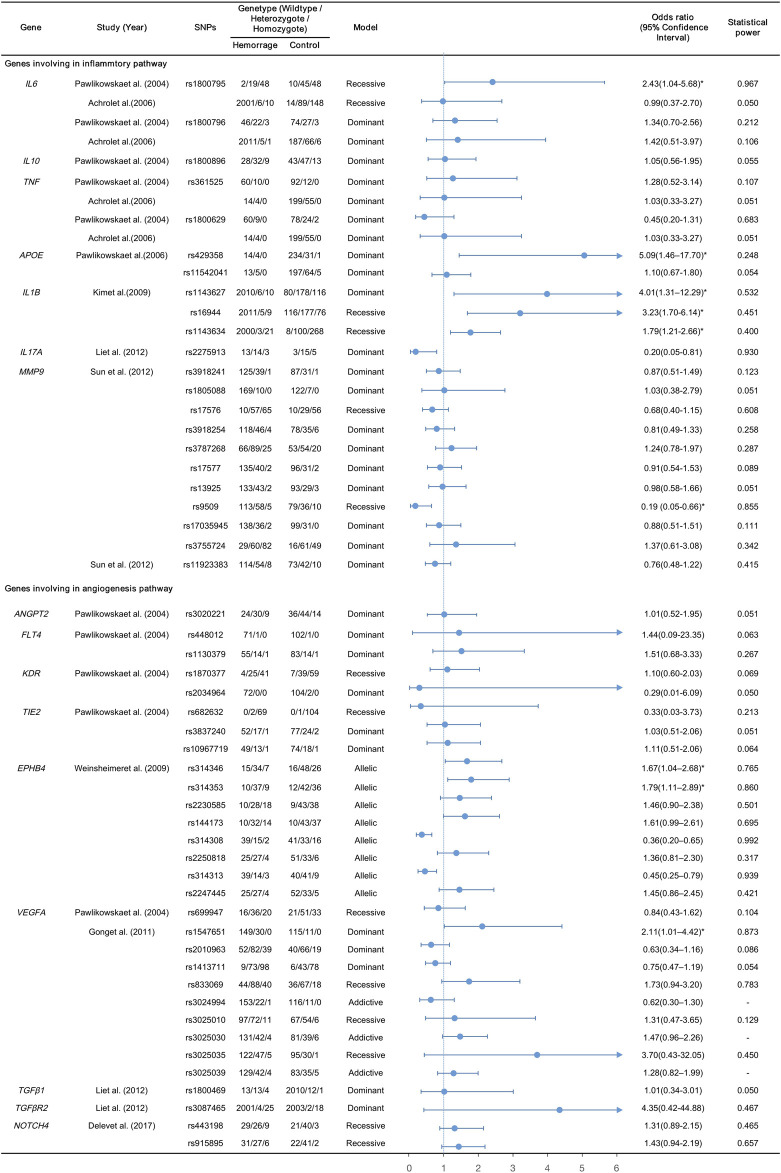
Summary and forest plots for the reported variants; *, calculated by multivariant analysis.

The nine CGASs evaluated SNPs in seven genes (*IL6*, *IL10*, *TNF, APOE*, *IL1B*, *IL17A*, *MMP9*) of the inflammatory pathway and nine genes (*ANGPT2*, *FLT4*, *KDR*, *TIE2*, *EPHB4*, *VEGFA*, *TGFβ1*, *TGFβR2*, *NOTCH4*) of the angiogenic pathway (Table 1; Figure 2). Seven SNPs in five inflammatory genes were reported to be significantly associated with bAVM rupture, including *APOE* rs429358 (OR, 5.09; 95% CI, 1.46–17.70), *IL1B* rs1143627 (OR, 4.01; 95% CI, 1.31–12.29), and *IL17A* rs2275913 (OR, 0.20; 95% CI, 0.05–0.66) in dominant models, and *IL6* rs1800795 (OR, 2.43; 95% CI, 1.04–5.68), *IL1B* rs16944 (OR, 3.23; 95% CI, 1.70–6.14), *IL1B* rs1143634 (OR, 1.79; 95% CI, 1.21–2.66), and *MMP9* rs9509 (OR, 0.19; 95% CI, 0.05–0.66) in recessive models. Five SNPs in two angiogenic genes were discovered to contribute to bAVM-related hemorrhage: *VEGFA* rs1547651 (OR, 2.11; 95% CI, 1.01–4.42) in dominant models, and rs314346 (OR, 1.67; 95% CI, 1.04–2.68), rs314353 (OR, 1.79; 95% CI, 1.11–2.89), rs314308 (OR, 0.36; 95% CI, 0.20–0.65), and rs314313 (OR, 0.45; 95% CI 0.25–0.79) in allelic models of *EPHB4*.

### 3.3 Methodological quality assessment

The statistical power of each polymorphism was calculated using the respective genotype model. The powers of the three SNPs in *VGEFA* could not be calculated as their ORs and 95% CIs were derived using additive models, with *α* set at 0.05. The 44 SNPs that were not associated with bAVM-related hemorrhage failed to reach sufficient statistical power (range, 0.050–0.783). Among the remaining 12 candidate variants, *APOE* rs429358 (power, 0.248), *IL1B* rs1143627 (power, 0.532), *IL1B* rs16944 (power, 0.451), *IL1B* rs1143634 (power, 0.400) and *EPHB4* rs314346 (power, 0.765), there were risks of false negatives. Only seven SNPs (12.5%) demonstrated powers greater than 0.80, with *IL6* rs1800795, *IL17A* rs2275913, *EPHB4* rs314308, and rs314313 reaching powers >0.90.

**FIGURE 3:**
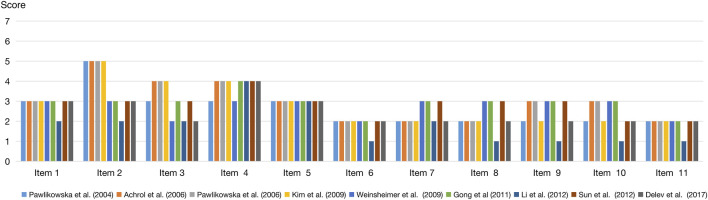
Methodological quality assessment using Q-genie tool (version 1.1), including 11 items: Item 1, rationale for study; Item 2, selection and definition of outcome of interest; Item 3, selection and comparability of comparison groups; Item 4, technical classification of the exposure; Item 5, non-technical classification of the exposure; Item 6, other sources of bias; Item 7, sample size and power; Item 8, a priori planning of analyses; Item 9, statistical methods and control for confounding; Item 10, testing of assumptions and inferences for genetic analyses; and Item 11, appropriateness of inferences drawn from results.

Although two-thirds of the included studies were judged to be of high quality (average 6.1 stars) after assessment by NOS (Table 2), all of them were classified as high-bias studies using the Q-Genie tool (Figure 3 **)**. Regarding methodology, most studies performed well in identification of patients with bAVM, as well as the methods for identification of genetic variants. However, there was still much room for improvement in the following areas: representativeness for the gene and SNP selection procedure as well as patients with bAVM; comparability between hemorrhagic and non-hemorrhagic individuals as confounding factors, as these were hardly taken into sufficient consideration; and a longer follow-up period in cohort studies.

**TABLE 2:** Summary of the included genetic studies on brain arteriovenous malformation related hemorrhage.

Author (Year)	Study design	Selection						Comparability		Exposure*			Outcome#			Total score
		Case definition*	Case or exposed cohort representativeness*^#^	Selection of controls or unexposed cases*^#^	Definition of controls*	Ascertainment of exposure^#^	Demonstration of unhappened outcome of interest^#^	Important factors*^#^	Additional factors*^#^	Ascertainment*	Same method of ascertainment for cases and controls*	Non-response rate*	Assessment of outcome^#^	Follow-up period^#^	Adequacy of follow up of cohorts^#^	
Pawlikowska et al. (2004)	Case- control	According to cerebral angiography by an attending interventional neuroradiologist. (*)	Consecutively recrruited in single medical center. (*)	Unruptured bAVM patients recruited in the same medical center. (*)	Patients had clinical presentation with ICH and signs of new intracranial hemorrhage on CT or MRI. (*)	—	—	Including race and gender, but not diagnosed age. (-)	Including bAVM size and venous drainage. (*)	Template-directed dye-terminator incorporation assay with fluorescence polarization detection. (*)	Yes(*)	NA (-)	—	—	—	7
Achrol et al. (2006)	Cohort	—	Consecutively recrruited in two medical center. (*)	Unruptured bAVM patients recruited in the same medical centers. (*)	—	Template-directed dye-terminator incorporation assay with fluorescence polarization detection. (*)	New ICH (*)	Including diagnosed age, race and gender. (*)	Including bAVM size, venous drainage and initial ICH presentation. (*)	—	—	—	Patients had clinical presentation with ICH and signs of new intracranial hemorrhage on CT or MRI. (*)	Median follow-up time was 0.31 years and interquartile range was 1.40 years (-)	NA (-)	7
Pawlikowska et al. (2006)	Cohort	—	Consecutively recrruited in two medical center. (*)	Unruptured bAVM patients recruited in the same medical centers. (*)	—	Template-directed dye-terminator incorporation assay. (*)	New ICH (*)	Including diagnosed age, race and gender. (*)	Including bAVM size, venous drainage and initial ICH presentation. (*)	—	—	—	Patients had clinical presentation with ICH and signs of new intracranial hemorrhage on CT or MRI. (*)	Median follow-up time was 0.30 years and interquartile range was 1.36 years (-)	NA (-)	7
Kim et al. (2009)	Cohort	—	Consecutively recrruited in two medical center. (*)	Unruptured bAVM patients recruited in the same medical centers. (*)	—	Template-directed dye-terminator incorporation assay with fluorescence polarization detection. (*)	New ICH (*)	NA (-)	NA (-)	—	—	—	Patients had clinical presentation with ICH and signs of new intracranial hemorrhage on CT or MRI. (*)	Mean follow-up time was 3.1 years and standard deviation was 7.5 years (-)	NA (−)	5
Weinsheimer et al. (2009)	Case- control	According to cerebral angiography by an attending interventional neuroradiologist, or checked in records by a trained medical records analyst. (*)	Consecutively recrruited in two medical center. (*)	Unruptured bAVM patients recruited in the same medical centers. (*)	Patients had clinical presentation with ICH and signs of new intracranial hemorrhage on CT or MRI. (*)	—	—	Including race and gender, but not diagnosed age. (-)	Including venous drainage, but not bAVM size. (-)	Beckman Coulter SNPstream 48plex technology or by template-directed primer extension with fluorescence polarization detection. (*)	Yes (*)	NA (-)	—	—	—	6
Gong et al. (2011)	Case- control	According to pathology or angiography. (*)	Consecutively recrruited in single medical center. (*)	Unruptured bAVM patients recruited in the same medical center. (*)	Patients had clinical presentation with ICH and signs of new intracranial hemorrhage on CT or MRI. (*)	—	—	Including diagnosed age, race, and gender. (*)	Neither bAVM size and venous drainage were not matched. (-)	Allele-specific MALDI-TOF mass spectrometry assay with MassARRAY iPLEX platform. (*)	Yes (*)	NA (-)	—	—	—	7
Li et al. (2012)	Case control	According to angiography. (*)	NA (−)	Unruptured bAVM patients recruited in the same medical center. (*)	Patients had clinical presentation with ICH and signs of new intracranial hemorrhage on CT or MRI. (*)	—	—	Including race, but not diagnosed age and gender. (-)	Neither bAVM size and venous drainage were not matched. (-)	Sanger sequencing. (*)	Yes (*)	NA (−)	—	—	—	5
Sun et al. (2012)	Case- control	According to angiography. (*)	Consecutively recrruited in single medical center. (*)	Unruptured bAVM patients recruited in the same medical center. (*)	Patients had clinical presentation with ICH and signs of new intracranial hemorrhage on CT or MRI. (*)	—	—	Including race and gender, but not diagnosed age. (-)	Neither bAVM size and venous drainage were not matched. (-)	Sequenom MassARRAY SNP genotyping platform. (*)	Yes (*)	NA (-)	—	—	—	6
Delev et al. (2017)	Case- control	According to pathology or angiography. (*)	NA (-)	Unruptured bAVM patients recruited in the same medical center. (*)	NA (-)	—	—	Including diagnosed age, race and gender. (*)	Including venous drainage, but not bAVM size. (-)	Big Dye Terminator Cycle Sequencing V1.1 Ready Reaction kit and automated ABI-3100 DNA sequencer. (*)	Yes (*)	NA (-)	—	—	—	5

*, standards of NOS scale for case-control studies; #, standards of NOS scale for corhot studies; ICH, intracranial hemorrhage; CT, computed tomography; MRI, magnetic resonance imaging; SNPs, single nucleotide polymorphisms; (*), studies won the star in each item of NOS; (-), studies did not win the star in each item of NOS.

## 4 Discussion

This cross-sectional study systematically reviewed the published studies on genetic factors associated with bAVM-related hemorrhage and identified nine correlative research works, which were examined for statistical power as well as methodological quality using the NOS and Q-Genie tool. This led to the identification of statistically significant association between bAVM-related hemorrhage and twelve heritable variants of seven genes (*IL6*, *APOE*, *IL1B*, *IL17A*, *MMP9*, *EPHB4*, and *VEGFA*) involved in the inflammatory and angiogenic signaling pathways. After methodological assessment, limitations were noted in the study designs of the included research works, indicating that the quality and reliability of these studies needed to be improved.

The biological functions of the identified candidate genes are known to be involved in inflammatory and angiogenic signaling pathways. We summarized that SNPs of five inflammatory genes (*IL6*, *APOE*, *IL1B*, *IL17A*, *MMP9*) were reported to be associated with bAVM-related hemorrhage. Additionally, it has been reported that these genes can increase the expression of inflammatory cytokines in bAVM tissues, leading to endothelial dysfunction and malformation of vasculature (Krithika and Sumi, 2021). It has been shown that inflammatory infiltration can be observed even in unruptured bAVM lesions, proving the role of inflammation in the development and rupture of the disease (Liu et al., 2022). As the disease progresses, endothelial lesions would weaken the vasculature, and once patients are exposed to a trigger, an acute hemorrhage event occurs. Many studies have investigated genes involved in angiogenesis signaling and their associations with bAVM(18). The reported genes (*EPHB4*, *VEGFA*, and also *MMP9*) are involved in the signaling of vascular endothelial growth factor (VEGF), a representative signaling molecule of angiogenesis. VEGF is highly expressed in endothelial cells of bAVM, especially in ruptured bAVM lesions (McDonald et al., 2015). Activation of this pathway could promote endothelial cell migration and recruitment of smooth muscle cells, resulting in pathological angiogenesis (Liu et al., 2022). A recent study using mouse bAVM models demonstrated that an elevated VEGF level could contribute to bAVM hemorrhage by exposure to variable degrees of higher intraluminal flow and hypertension in the venous system (Cheng et al., 2019).

Almost all of the included studies discussed the limitation of their small sample size (mean, 223.78; standard deviation, 106.40). Only seven (12.50%) of the 52 calculated statistical powers for each SNP were more than 0.80, indicating a lower risk of Type II error. Therefore, a large study cohort would be preferable in order to achieve a statistical power 0.80 or more and to detect relatively reliable association of bAVM-related hemorrhage with the genetic variants of bAVM. All nine included studies used a CGAS design for the genotyping of individuals. The selection of genes and SNPs was based on their known biological functions. Four studies selected tagging SNPs from the HapMap project data (http://www.hapmap.org), which takes the initiative of genotyping human populations around the world and narrows down the significant loci associated with reviewed diseases (Patnala et al., 2013). The other five studies chose SNPs located in the promoters or exomes of inflammatory/angiogenesis genes to explore their associations with bAVM-related hemorrhage. However, the researchers ignored the possibility that several genes with unknown functions may also be involved in the pathogenic process of bAVM. Thus, with the development of sequencing platforms and techniques, advanced methods should be used to detect the increasing number of genetic associations of bAVMs involving GWAS and WES (McCarthy et al., 2008; Wijmenga and Zhernakova, 2018; Tam et al., 2019). After obtaining sufficiently large whole-genome sequencing datasets, machine learning can be a practical tool to extract key information efficiently (Powell et al., 2021). Based on a variatiy of statistical approaches and biological processes of genes involved in development, signaling, disease, and homeostasis, unsupervised machine learning approaches are not biased by allele frequencies, even without reliance on prior knowledge, to identify heretofore unrecognized genetic risk factors (Powell et al., 2021; Mizikovsky et al., 2022). In addition, the representativeness of the included patients with bAVM should be mentioned. Although most of the studies recruited their cohorts consecutively, the limited number of patients who only came from one or two medical centers failed to represent the populations of their regions or countries, contributing to selection bias. Therefore, we advocate for multi-center cohorts of bAVM patients with large sample sizes to improve the representativeness of the studies.

Another issue that demands greater attention is how to avoid confounding risk factors to ensure comparability. During the study design process, four studies used the strategy of matching and taking baseline characteristics (sex, age, and race) into consideration to reduce confounding bias and improve reliability (Zeldow and Hatfield, 2021). Seven studies were able to achieve statistically significant results by performing multivariate analysis to adjust for not only for the above mentioned baseline characteristics, but also for the identified morphological risk factors, such as the size and draining veins of bAVM. Three included cohort studies were conducted by the same research team; two of which considered prior hemorrhage an independent risk and confounding factor to assess the associations between genetic factors and bAVM-related hemorrhage, therefore, only new hemorrhagic events were evaluated their association with genetic risk factors, but not the overall risk of hemorrhage. Hence, it would be preferable to recruit patients with unruptured bAVM in future cohort studies. Additionally, it should be noted that the follow-up period was too short (median follow-up period was 4 months in two studies, and average follow-up was 3.1 years in one) to obtain reliable results, since some patients may have experienced hemorrhagic events after the last follow-up, resulting in a misclassification. Furthermore, the researchers used the time point and clinical information of the last follow-up, instead of regarding them as lost subjects. Calculating the rate of loss to follow-up and evaluating the adequacy of the follow-up stage are challenging tasks. Thus, in order to improve the research quality and accuracy of results, it is necessary to set an appropriate follow-up duration so that outcome events can be observed as much as possible, while preventing excessive environmental confounding factors.

To promote the genetic study on bAVM-related hemorrhage, the methodological issues in other diseases are also worth referring to. Two strategies are usually performed to identify the disease-causing variants: sequencing affected individuals with a family history and extreme-trait sequencing (Cirulli and Goldstein, 2010). The first strategy is widely adopted for rare neurological and cerebrovascular diseases, such as moyamoya disease and hereditary hemorrhagic telangiectasia (Liu et al., 2011; Benzinou et al., 2012). It is easier to detect co-effected members within families to identify overlapping variants. Considering a relatively low incidence of bAVM and its related hemorrhage, as well as its rare familial cases, it is of more value for clinicians to consciously collect these individuals for genetic study in their clinical practice (Chen et al., 2020). Extreme-trait sequencing is based on a hypothesis that the enrichment of rare variants would result in an extreme phenotype (Cirulli and Goldstein, 2010). Thus, it could be efficient to filter damaging variants by recruiting individuals with extreme-traits, including: large lesions, epileptic symptoms or an early onset of hemorrhage.

This study had several limitations. First, owing to the low incidence of bAVM, only a small number of studies with small sample sizes and insufficient statistical powers were included in the assessment. Second, the included studies published only in English or Chinese, resulting in a reduction of representativeness. Third, we calculated the statistical powers assuming *α* = 0.05, while the results would be more reliable by using the Bonferroni correction and multiple comparisons similar to those used in GWAS. However, the sample sizes of the existing studies did not reach a statistical significance. Thus, multi-center studies are warranted to increase the sample size and improve comparability.

## 5 Conclusion

Several genes were identified to be associated with bAVM-related hemorrhage, including: *IL6*, *IL17A*, *MMP9*, *VEGFA*, *EPHB4*. Efforts are needed to investigate the mechanism of bAVM-related hemorrhage in the future. We call for the establishment of regional alliances and rare disease banks in order to perform a multicenter prospective cohort study with a large sample size of patients with bAVM and adequate follow-up. Familial and extreme-trait cases are precious genetic resources for high-throughput sequencing. Furthermore, it may be more efficient to investigate genetic risks capitalizing on machine learning, Multi-omic systems-based approaches should be utilized to uncover the roles of candidate genes in bAVM development. Designers and researchers should strive to improve the methodological quality of their studies according to the demands of assessment tools, especially avoiding confounding risk factors, to ensure comparability.

## Data Availability

The original contributions presented in the study are included in the article/supplementary material, further inquiries can be directed to the corresponding authors.

## Abbreviations

bAVM, brain arteriovenous malformation; CGAS, candidate gene association studies; CNKI, China National Knowledge Infrastructure; GWAS, genome-wide association studies; NOS, Newcastle–Ottawa scale; OR, odds ratio; SNPs, single nucleotide polymorphisms; VEGF, vascular endothelial growth factor; WES, whole exome sequencing; 95% CI, 95% confidence interval.
